# Interventional low‐dose azithromycin attenuates cigarette smoke‐induced emphysema and lung inflammation in mice

**DOI:** 10.14814/phy2.14419

**Published:** 2020-07-11

**Authors:** Matthew G. Macowan, Hong Liu, Marianne D. Keller, Miranda Ween, Rhys Hamon, Hai B. Tran, Sandra Hodge

**Affiliations:** ^1^ Department of Thoracic Medicine Royal Adelaide Hospital Adelaide SA Australia; ^2^ Adelaide Medical School University of Adelaide Adelaide SA Australia; ^3^ Department of Immunology and Pathology Monash University Melbourne VIC Australia; ^4^ Preclinical, Imaging and Research Laboratories (PIRL) South Australian Health and Medical Research Institute (SAHMRI) Adelaide SA Australia; ^5^ Sydney School of Veterinary Science Faculty of Science University of Sydney Sydney NSW Australia; ^6^ Centre for Cancer Biology University of South Australia and SA Pathology Adelaide SA Australia

**Keywords:** chronic obstructive pulmonary disease, COPD, emphysema, in vivo imaging, magnetic resonance imaging, micro‐CT, mouse model

## Abstract

Cigarette smoke (CS)‐induced emphysema is an important contributor to chronic obstructive pulmonary disease (COPD). We have shown the efficacy of azithromycin in reducing airway inflammation in COPD and in reducing exacerbations in severe asthma; however, the effects of long‐term azithromycin on emphysema development have not been shown. We employed live animal imaging to monitor emphysema‐like development and the effects of interventional azithromycin treatment in CS‐exposed mice. BALB/c mice (female, 10 weeks; *n* = 10) were exposed to CS for 1 hr twice daily, 5 days/week, and for 12 weeks (CS). Half were cotreated with low‐dose azithromycin during weeks 7–12 (CS + Azi; 0.2 mg kg^−1^ day^−1^). Microcomputed tomography (CT) and magnetic resonance imaging (MRI) scans were acquired longitudinally. Histological examinations were performed post mortem (mean linear intercept (Lm) and leukocyte infiltration). CS increased median Lm (CS: 42.45 µm versus control: 34.7 µm; *p* = .0317), this was recovered in CS + Azi mice (33.03 µm). Average CT values were reduced in CS mice (CS: −399.5 Hounsfield units (HU) versus control: −384.9 HU; *p* = .0286) but not in CS + Azi mice (−377.3 HU). CT values negatively correlated with Lm (*r* = −.7972; *p* = .0029) and T_2_‐weighted MRI (*r* = −.6434; *p* = .0278). MRI also showed significant CS‐induced inflammatory changes that were attenuated by azithromycin in the lungs, and positively correlated with Lm (*r* = .7622; *p* = .0055) and inflammatory foci counts (*r* = .6503; *p* = .0257). Monitoring of emphysema development is possible via micro‐CT and MRI. Interventional azithromycin treatment in CS‐exposed mice attenuated the development of pulmonary emphysema‐like changes.

## Graphical abstract

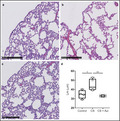

## 1 INTRODUCTION

Emphysema, alongside chronic bronchitis, is the most common condition in chronic obstructive pulmonary disease (COPD), for which cigarette smoke (CS) is the most common causative factor. The World Health Organization expects COPD to become the third leading cause of death worldwide by 2030 (Alwan, (2010); Quaderi & Hurst, 2018). As COPD is incurable, and current therapies primarily target only disease symptoms, there is an urgent need for novel treatment strategies. In contrast to healthy airways, COPD patients exhibit chronic lung inflammation and loss of structural integrity in both the large and small airways (Jeffery, 2001). Emphysema is characteristic of CS‐induced COPD, and involves the destruction of lung parenchyma and permanent enlargement of the alveolar spaces distal to the terminal bronchioles (Jeffery, 2001; Snider, Kleinerman, Thurlbeck, & Bengali, 1985).

Azithromycin is a macrolide antibiotic derived from erythromycin that is known to be effective against many Gram‐negative bacteria, including some of clinical relevance in COPD such as (non‐typeable) *Haemophilus influenzae* (Hodge et al., 2017; Peters, Friedel, & McTavish, 1992). In addition to its antimicrobial function, it has been shown to have anti‐inflammatory activity which may contribute to its observed therapeutic effects in asthma and COPD patients (Segal et al., 2017). We have previously shown that low‐dose azithromycin treatment in COPD patients improves macrophage clearance of apoptotic cells and bacteria, and reduces airway inflammation (Albert et al., 2011; Hodge et al., 2008; Hodge & Reynolds, 2012; Pomares et al., 2011). Others have reported that long‐term azithromycin treatment also decreased COPD exacerbations and improved quality of life (Albert et al., (2011); Pomares et al., 2011). We have further shown its efficacy in reducing exacerbations in severe asthmatic patients who can also suffer from chronic lung inflammation (Gibson et al., 2017).

There have been limited studies of the effects of macrolide antibiotics in emphysematous animal models. One study reported the success of clarithromycin in preventing CS‐induced emphysema‐like changes in a mouse model (Nakanishi et al., 2009), while prior work in a rat model of CS‐induced COPD showed that daily intragastric administration of azithromycin (50 mg/kg) attenuated emphysema‐like changes and lung function (FEV_1_) loss, and reduced inflammatory cell numbers (Wan et al., 2015).

However, no study has investigated whether a macrolide can attenuate development of emphysema‐like changes when administered at a low dosage following CS‐induced development of an inflammatory, yet pre‐emphysematous lung environment. This model is representative of a treatment that prevents emphysema development in a smoker experiencing minor lung distress. Therefore, in this study, we utilized live animal imaging to monitor the inflammatory lung environment and emphysema‐like changes development in CS‐exposed mice treated with interventional inhaled azithromycin.

## 2 METHODS

### 2.1 Cigarette smoke exposure and Azithromycin treatment

The protocol was adapted from our previous publications (Beckett et al., 2013; Hodge et al., 2010, 2011; Mukaro et al., 2013). Female BALB/c mice (10 weeks old; CS and CS + Azi: *n* = 5 mice/group) were exposed to mainstream CS from nine research cigarettes (1R6F reference cigarette, University of Kentucky, KY, USA) for 1 hr, twice daily, 5 days/week, for 12 weeks. The protocol utilized the inExpose system and whole‐body chambers (Scireq Inc, QC, Canada), control mice (*n* = 5) were exposed to fresh air in an adjacent inExpose chamber. inExpose puff profile was configured to deliver 2 L/min fresh air, with an ISO standard CS puff once every 30 s. Cigarettes were burnt to 3 mm from the tipping paper. During weeks 7–12, the CS + Azi cohort were exposed to 0.2 mg kg day^−1^ of azithromycin via nebulization [azithromycin (Sigma Aldrich, MO, USA) dissolved in pH 6.0 PBS] into the inExpose whole‐body chamber once per day following their second CS session.

Animals were housed within a specific pathogen‐free barrier facility, in separate cages for each cohort, with a temperature range 18–24°C, 12:12 hr light‐dark cycle, and few regular chow ad‐libitum. The experiments were approved by SAHMRI Animal Ethics Committee (SAM361) and conducted within the National Health and Medical Research Council of Australia guidelines on animal experimentation.

### 2.2 Histological processing

Lung tissues were perfused with 10% formalin solution (Chem‐Supply, SA, Australia), fixed overnight then embedded. Five µm sections were stained with H&E, then imaged at 20× objective resolution using a NanoZoomer 2.0‐HT (Hamamatsu Photonics, Japan), and viewed using NDP.view 2 software (Hamamatsu) for further analysis.

### 2.3 Calculation of mean linear intercept

Mean linear intercept (Lm) values advised alveolar size and the extent of emphysematous changes (Aoshiba, Yokohori, & Nagai, 2003; Nadziejko, Fang, Bravo, & Gordon, 2007). To calculate Lm, 5 nonoverlapping regions/mouse were systematically acquired (20× objective resolution) from each of three sections approximately 120 µm apart. These images were analyzed via Fiji (Schindelin et al., 2012) using an in‐house script. Briefly, a) the image size was calibrated; b) an overlay with parallel lines was added, and a selection created from the tissue components which were subtracted from the grid; c) the remaining lines (minimum length 10 µm) were automatically measured, and the average length returned, that is, Lm. This was repeated with both horizontal and vertical lines to avoid rotation bias. Only regions without bronchioles, large blood vessels (>50 µm), and other nonairway sections were selected.

### 2.4 Microcomputed tomography

Micro‐CT images were acquired via the Skyscan 1,176 (Bruker Physik GmbH, Germany) at weeks 0, 6, 8, 10, and 12 using a 55 kV source voltage, 455 µA source current, 0.7° rotation step, 200 ms exposure, and 18 µm pixel size; physiological monitoring mode was synchronized to chest movement. Ten‐minute average scan times resulted in an average whole‐body exposure of 155 mGy/scan. Mice were scanned supine and anesthetized with an isoflurane/oxygen mixture. Hounsfield units (HU) were calibrated prior to each imaging session by scanning a 50‐mL tube of distilled water.

Images were reconstructed between HU values of −1000 and 1,000 using NRecon (Bruker) with smoothing of 6, ring‐artifact reduction of 5, and beam‐hardening correction of 20%. Using DataViewer (Bruker), datasets were realigned, and the lung volume of interest dorsal on the sagittal plane to between the third and fourth sternebrae and between the T9 and T10 thoracic vertebrae was selected [to highlight emphysematous changes (Snider et al., 1985), and for its approximation to previous segmentation strategies in human studies (Hoesein et al., 2012; Lee, Kim, Kim, Ahn, & Kim, 2018)]. Images were analyzed in CTan (Bruker), and average lung HU values recorded.

### 2.5 Magnetic resonance imaging

Two T_1_‐weighted gradient echoes were acquired: (TR/TE = 300.9/6.21 ms; 1 NEX; vow = 80 mm^2^; slice thickness = 2 mm; <1 min), and a longer scan with higher resolution (TR/TE = 382.9/6.21 ms; 4 NEX; vow = 80 mm^2^; slice thickness = 2 mm; 6.5 min). Then, a T_2_‐weighted RARE spin‐echo sequence was acquired (TR/TE = 4,314.3/84 ms; 1 NEX; rare factor = 8; vow = 80 mm^2^; slice thickness = 2 mm; <2 min). ITK‐SNAP software (Yushkevich et al., 2006) was used for consequent segmentation and analysis of the scans. T_2_‐intensity ratios were calculated by dividing mean image intensity of the lungs (and kidneys) by the mean intensity of arm muscle in the same slices to normalize values.

### 2.6 Enumeration of leukocyte infiltration foci

To determine leukocyte infiltration, focal clusters of >20 infiltrating leukocytes were counted in NDP.view 2 (Hamamatsu). Entire lung sections were scored, and total area measured (3 sections/mouse; approximately 120 µm apart). Foci per 20 mm^2^ were calculated.

### 2.7 Statistical analyses

Kruskal–Wallis and Mann–Whitney *U*‐tests, or Wilcoxon signed rank tests were applied as appropriate. Spearman's rank‐order correlation was used to assess association between variables. Analyses were performed using GraphPad Prism 7 (GraphPad Software, CA, USA). Data are presented as median and range, unless otherwise specified. *p* < .05 statistically significant.

## 3 RESULTS

### 3.1 Animal welfare

No signs of stress were evident during handling or the CS exposure protocol in BALB/c mice. During the first 2 weeks, the majority of mice became visibly drowsy by the third or fourth cigarette, however, this effect lessened over time, as the mice became more accustomed to CS exposure. CS‐exposed mice were also more lethargic when returned to their cages, but appeared to recover fully within 1–2 hr. By approximately week 4 of exposure, mice were eager to enter the Scireq apparatus, presumably due to nicotine addiction; this was especially evident on Mondays following 2 days without cigarettes.

**FIGURE 1:**
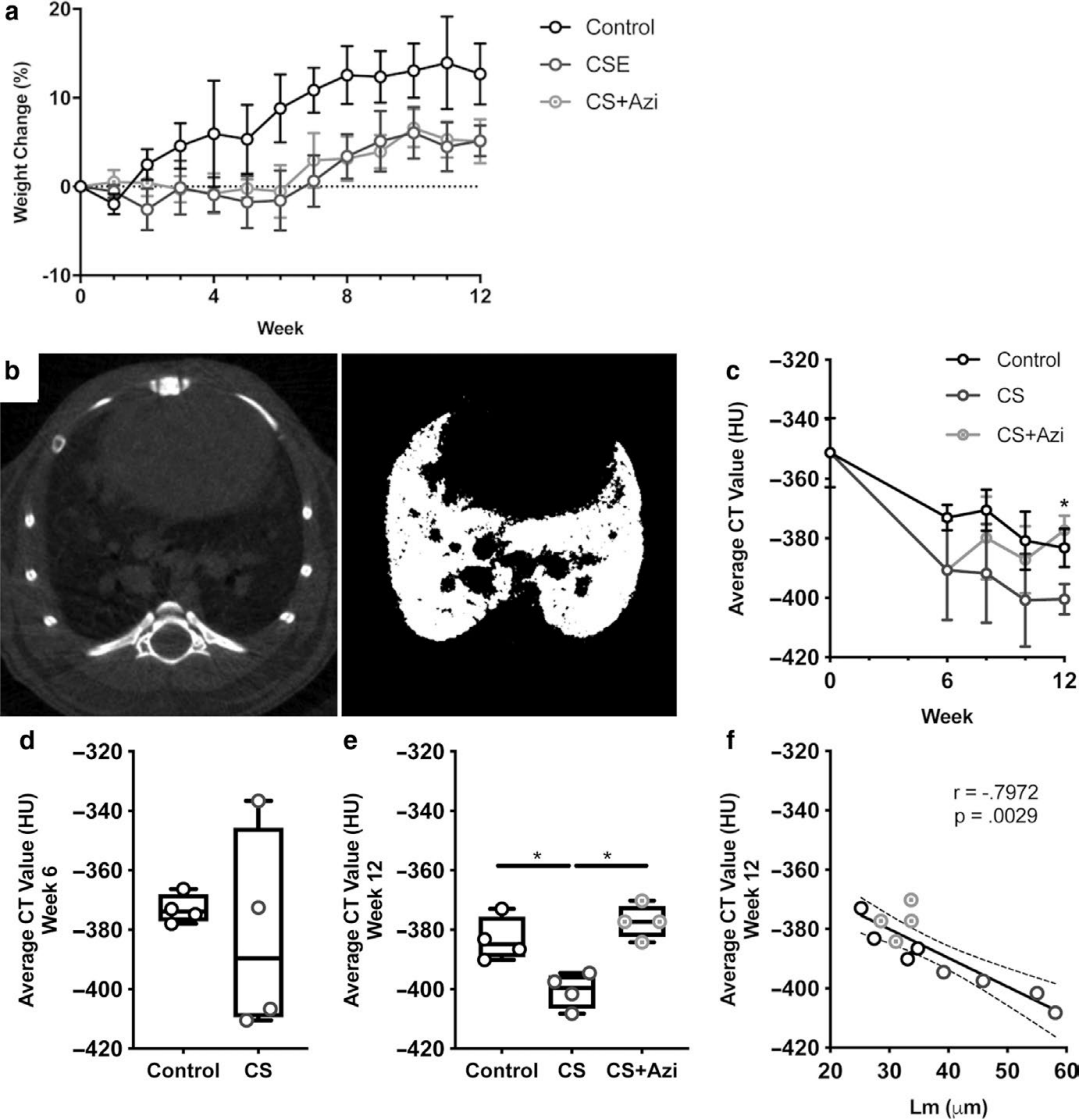
Emphysematous changes in CS‐exposed mice via micro‐CT analysis of the lower lung are attenuated by azithromycin. (a) Percentage changes in mouse weight over time. (b) Representative micro‐CT image slice of a mouse lung prior to processing (left) and postthresholding (right). (c) Time course of average Hounsfield unit (HU) values of the lower lungs (mean ± *SD*). (d) Average HU values at week 6. (e) Average HU values at week 12 prior to postmortem. (f) Correlation of week 12 average HU values with Lm. Data presented as median ± range (unless otherwise stated). * represents significance between cohorts, *p* < .05. Dotted lines show the 95% confidence interval

CS‐exposed mice failed to gain weight until week 7 (contrary to controls), although weight loss never exceeded 6% (Figure 1a); this is similar to a previous CS exposure study in BALB/c mice (Beckett et al., 2013). It should be noted that the CS‐exposed BALB/c mice used in this study became visibly stained by the CS, and therefore during grooming it is possible they were further exposed to tar and nicotine.

### 3.2 Histological assessment of alveolar destruction

**FIGURE 2:**
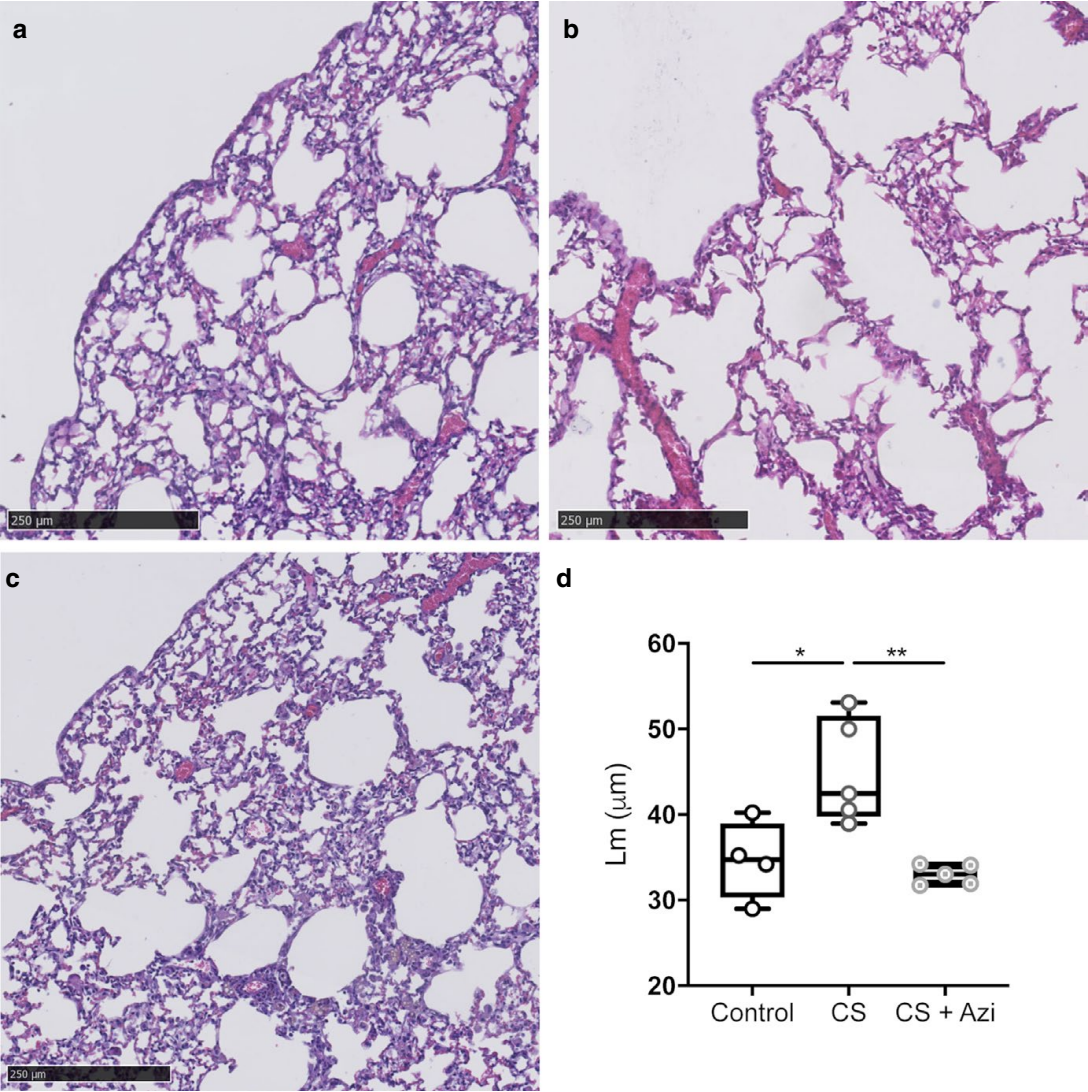
Assessment of emphysema‐like changes in the lungs. (a–c) Representative H&E‐stained lung sections at 12 weeks. (a) control, (b) CS‐exposed (CS), and (c) CS‐exposed + azithromycin treatment (CS + Azi). Scale bar is 250 µm. (d) Mean linear intercept (Lm) values were taken as a measure of alveolar size and emphysematous changes. Data represent the average Lm based on the measurements taken from 3 lung sections per mouse; median ± range. *n* = 4–5 mice/group. * represents significance between cohorts, *p* < .05; ** indicates *p* < .01

To examine the therapeutic potential of inhaled azithromycin treatment in reducing CS‐related emphysematous changes in the lungs, BALB/c mice were exposed to CS for 6 weeks, then CS ± azithromycin for an additional 6 weeks. Lm was significantly greater in CS mice compared with controls [20.09% increase; CS: 42.45µm (38.95 – 53.1), control: 34.7 µm (29 – 40.22); *p* = .0317] (Figure 2d). CS + Azi mice showed no difference in Lm compared with controls [33.03 µm (31.72 – 34.2)] (Figure 2d).

### 3.3 Micro‐CT analysis of the lungs

Average CT values (mean HU) from micro‐CT scans, restricted to the lower lung, acquired at weeks 0, 6, 8, 10, and 12 were analyzed. Kruskal–Wallis analysis of percentage changes revealed no significant intercohort differences at the time points examined (Figure 1c). Longitudinal tracking of absolute average HU in the lower lungs suggested significant decreases in density over the first 6 weeks in CS‐exposed mice [−399.1 HU (−412.4 – −336.6) in week 6 versus −357 HU (−370.2 – −301.9) in week 0; *p* = .0022]. Control mice also showed a decrease [−373.9 HU (−387 – −366.3) in week 6; *p* = .0132] (Figure 1c‐d). However, there was no significant differences between control and CS‐exposed cohorts at this time (*p* = .1535). Treatment of both CS‐exposed groups was identical during this period.

Significantly decreased average HU values in the lower lungs were observed in CS mice at week 12 [−399.5 HU (−408.2 – −394.5)] compared with both control mice [−384.9 HU (−390.1 – −373)] and CS + Azi mice [−377.3 HU (−384.2 – −370.2)] (Figure 1e; *p* = .0286). CS + Azi mice presented with average HU values similar to controls, and significantly greater than CS mice (*p* = .0286). Week 12 average HU values also showed significant negative correlation with Lm (Spearman *r* = −.7972; *p* = .0029) (Figure 1f).

### 3.4 MRI Analysis of the lungs

**FIGURE 3:**
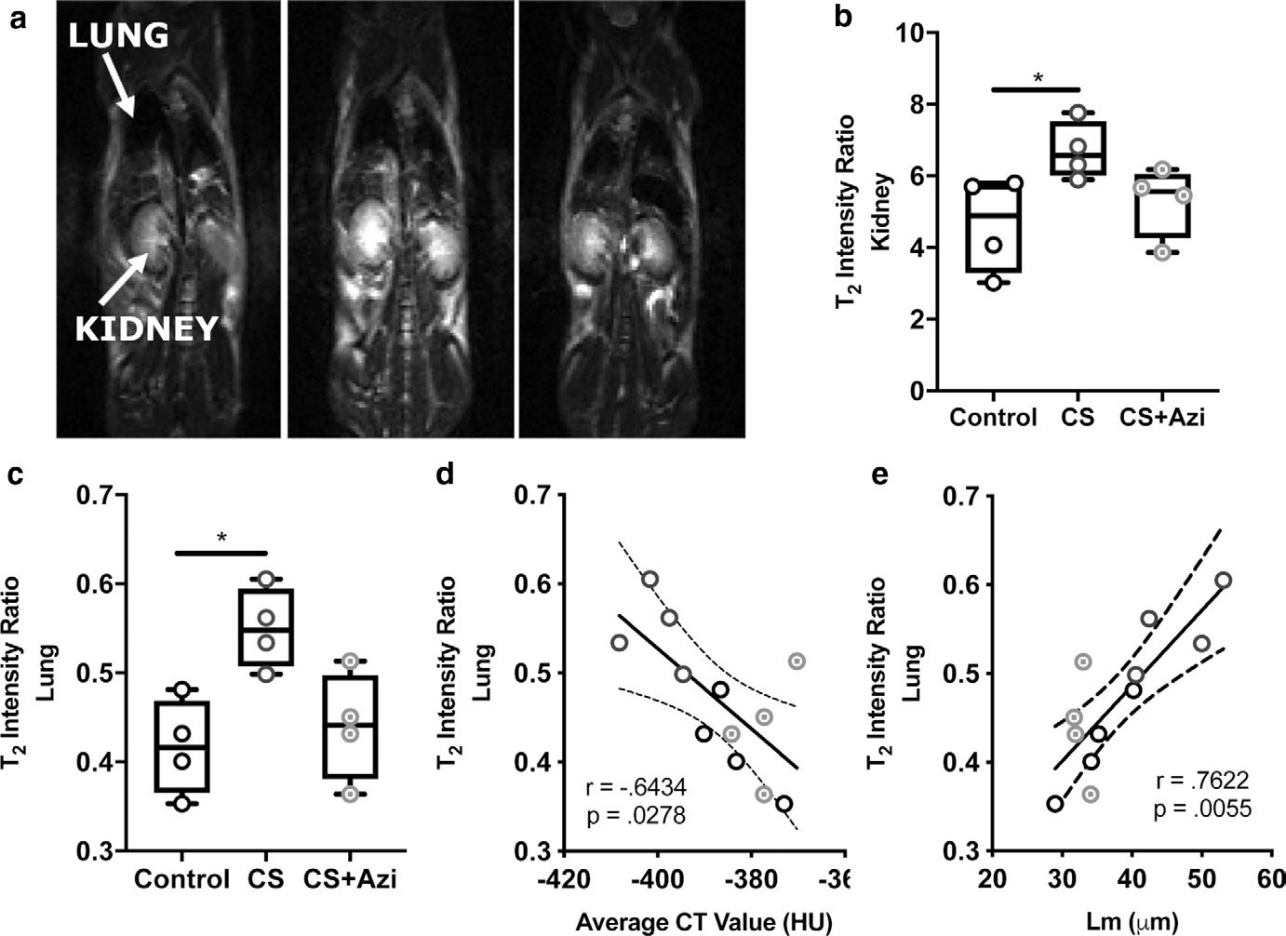
Inflammation in the lungs and kidneys of CS‐exposed mice is attenuated by azithromycin. (a) Representative coronal, T_2_‐weighted MR images of live mice. (b and c) Comparison of T_2_ intensity in the lungs and kidneys. (d) Correlation of T_2_‐weighted MRI intensity values of the lung and average CT value (HU). (e) Correlation of T_2_‐weighted MRI intensity values of the lung and Lm. Data presented as median ± range. Dotted lines show the 95% confidence interval. * represents significance between cohorts, *p* < .05. Dotted lines show the 95% confidence interval. *n* = 4 mice/group

MRI examination of lung inflammation revealed a 27.28% increase in T_2_‐weighted signal intensity ratio in CS mice (*p* = .0286), while CS + Azi mice exhibited nonsignificant differences from controls (Figure 3c). Similar analysis of the kidneys was also performed to detect inflammatory changes elsewhere in the body; T_2_ signal intensity was increased by 29.3% in CS mice (*p* = .0286) (Figure 3b). These MRI values negatively correlated with average HU values (Spearman *r* = −.6434; *p* = .0278) (Figure 3d), while showing significant positive correlation with Lm (Spearman *r* = .7622; *p* = .0055) (Figure 3e).

### 3.5 Perivascular and periepithelial leukocyte infiltration

**FIGURE 4:**
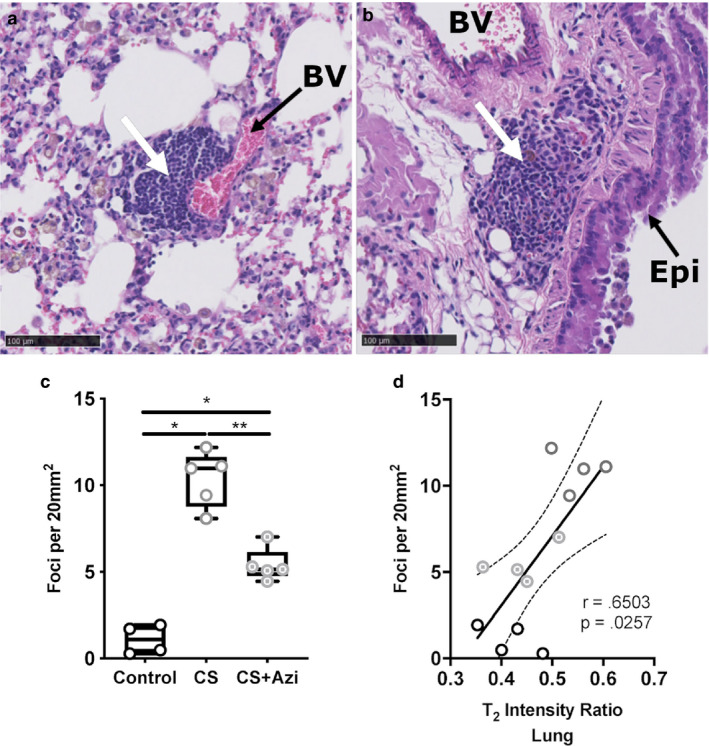
CS‐exposed mice showed increased leukocyte infiltration into lung tissue that was attenuated by azithromycin. (a and b) H&E‐stained lung sections from CS‐exposed mice showing examples of leukocyte foci (white arrows) (a) near blood vessels (BV) and (b) airway epithelia (Epi). (c) Fold change in the number of leukocyte foci in the lungs. (d) Correlation between T_2_‐weight MRI intensity values and leukocyte foci in the lung. Data presented as median ± range. Dotted lines show the 95% confidence interval. * represents significance between cohorts, *p* < .05; ** indicates *p* < .01. Dotted lines show the 95% confidence interval. *n* = 4–5 mice/group

Leukocyte infiltration foci (Figure 4a‐b) were increased 9.98‐fold in CS mice [10.98 foci/20 mm^2^ (8.079–12.180) versus 1.1 foci/20 mm^2^ (0.277–1.927) in controls; *p* = .0159] and 4.7‐fold in CS + Azi mice [5.147 foci/20 mm^2^ (4.460 – 7.014); *p* = .0159] compared with controls (Figure 4c). CS + Azi mice showed significantly fewer infiltration foci than CS mice (2.13‐fold fewer; *p* = .0079). Numbers of leukocyte infiltration foci present in the lungs showed significant positive correlation with MRI T_2_ signal intensity ratio (Spearman *r* = .6503; *p* = .0257) (Figure 4d).

## 4 DISCUSSION

Emphysema and increased inflammation are two components characteristic of the COPD phenotype. Emphysema severity is highly variable among COPD patients (even those at the same stage of disease progression) and increased severity is associated with poorer quality of life (Makita et al., 2007). We and others have previously highlighted the potential of macrolide antibiotics (viz., azithromycin) in COPD for reducing inflammation (Zarogoulidis et al., 2012) and pulmonary exacerbations (Albert et al., 2011; Pomares et al., 2011), improving bacterial clearance (Hodge et al., 2008; Hodge & Reynolds, 2012), and increasing the phagocytosis of apoptotic airway cells, that is, efferocytosis (Hodge et al., 2006). The macrolide antibiotic clarithromycin has also been shown to prevent CS‐induced emphysema‐like changes in mice via oral administration at doses ranging from 25 to 100 mg/kg twice daily (Nakanishi et al., 2009).

While these represent promising findings, there are concerns regarding the potential for selection of resistant bacteria following prolonged macrolide use at antibiotic concentrations (Taylor et al., 2019; Zarogoulidis et al., 2012). Our previous study investigating long‐term, low‐dose azithromycin use in asthma patients (500 mg tablets three times per week for 48 weeks) found a nonsignificant increase in sputum isolation of azithromycin‐resistant organisms compared with placebo patients [19 (50%) versus 12 (29%) of 42 organisms; *p* = .062] (Gibson et al., 2017). Another recent study using the same treatment in asthma patients reported a significant increase in azithromycin‐resistant bacterial isolates and overall expression of antibiotic resistance genes in the lungs, however, it noted that this was not associated with increased isolation of pathogenic species (Taylor et al., 2019). In an attempt to minimize the selection pressure while retaining its immunomodulatory effects, this study treated CS‐exposed mice daily with low‐dose azithromycin (0.2mg/kg) via inhalation of nebulized solution to target the lungs (as opposed to oral administration). This low dosage was chosen for its equivalence to our previous work both in vitro (Hodge et al., 2006, 2017) and in a human phase II study in COPD, where low‐dose azithromycin [250 mg orally for 5 days, then twice weekly (total of 12 weeks)] demonstrated anti‐inflammatory effects and improved alveolar macrophage phagocytic and efferocytic function(Hodge et al., 2008, 2017; Hodge & Reynolds, 2012).

Induction of emphysema‐like changes was confirmed in our CS mice through Lm measurements. The observed increase of approximately 20% is comparable with prior work in BALB/c and C57BL/6 mice, and reported to be indicative of emphysema in these animals (Beckett et al., 2013; Nadziejko et al., 2007; Nakanishi et al., 2009). Nose‐only exposure of BALB/c mice to CS has been shown previously to induce hallmark features of human COPD including increased Lm after 8–12 weeks (Beckett et al., 2013). We therefore present similar results at 12 weeks using less intense delivery via whole‐body exposure chamber. We also found low‐dose interventional azithromycin treatment attenuated Lm increase, that is, alveolar destruction, to control levels when administered during preemphysematous stages of development. Treatment with clarithromycin (50 mg/kg) from the commencement of CS exposure in C57BL/6 mice (Nakanishi et al., 2009), and also azithromycin (50 mg/kg) in Sprague–Dawley rats to a lesser degree (Wan et al., 2015), has been shown to achieve similar results. Importantly, we replicated these results using 0.4% of the dose by direct administration to the lungs via inhalation of nebulized azithromycin solution, and achieved comparable results despite 6 weeks of prior CS exposure alone. Nebulizers are already used in the treatment of COPD and acute asthma attacks, and their efficacy and ease of use has been shown previously (Ferguson et al., 2019; Hossein et al., 2016; Tashkin, 2016). Given that azithromycin is already approved for use in humans, and nebulizers are commonly used, this study warrants further investigation as a potential treatment avenue for pre‐COPD smokers, as there are currently no existing treatments for patients at the early stage of emphysematous lung disease. It should, however, be noted that CS exposure in mice for 12 weeks cannot fully recapitulate the changes seen in humans, and our findings require confirmation in human studies of early‐stage COPD development.

Time‐course analysis of average CT values (HU) of the lower lung was statistically nonsignificant between cohorts until week 12; CS + Azi mice showed lung density recovery to control levels by this time point. The significant decreases in lung density during the first 6 weeks were likely due to increases in mouse size and changing air space size (Kawakami, Paul, & Thurlbeck, 1984). In line with our findings, a previous study only showed significant changes in CS mice, compared with controls, at the 12‐week time point in C57BL/6 mice (Sasaki et al., 2015). The significant negative correlation between average HU values and Lm suggests that micro‐CT analysis may offer an effective means of estimating emphysema‐like development in live mice. Taking the CT and Lm results together, these findings point toward interventional azithromycin‐related protection in CS + Azi mice. Further studies and optimization in different strains of mice used for modeling COPD are required to determine whether micro‐CT analysis is feasible for the longitudinal tracking of emphysematous changes in the lungs.

The progressive airflow limitation observed in COPD patients results from a combination of emphysematous destruction of lung parenchyma and abnormal inflammatory responses; these changes are largely irreversible. Chronic inflammation in COPD patients persists despite cessation of smoking, and can increase further during exacerbations (Barnes, 2014, 2016). These inflammatory responses seen in COPD are characterized by increases in alveolar macrophages, neutrophils, and T lymphocytes recruited to the lung parenchyma from the circulatory system (Barnes, 2016; Suzuki et al., 2017; Wang, Xu, Meng, Adcock, & Yao, 2018).

Mucus production or edema is usually seen in cases of experimental lung inflammation and lung injury, causing increased T_2_‐weighted MRI signal intensity values to be observed (Beckmann et al., 2007). Therefore, the increased lung T_2_ signal intensity we report here in CS mice is likely the result of increased inflammation and fluid retention due to alveolar destruction and related elasticity loss (Beckmann et al., 2007), or potentially pulmonary infiltrates or thickening of the bronchial walls (Wielpütz & Kauczor, 2012). Importantly, we demonstrate that low‐dose azithromycin was able to reduce T_2_ signal intensity to near control levels following 6 weeks of treatment, despite the continuation of CS exposure throughout this period.

T_2_‐weighted single‐shot MRI with partial Fourier acquisition (HASTE) has been used in humans to identify lung infiltrates, mucus accumulation, and inflammatory thickening of the bronchi in obstructive lung diseases (Ley‐Zaporozhan, Puderbach, & Kauczor, 2008). Furthermore, a previous study in BALB/c mice investigating LPS‐induced inflammation demonstrated positive correlation between MRI signal intensity and histological analysis of lung injury (Conti et al., 2010), while an elastase‐induced model of emphysema in Brown Norway rats reported significant positive correlation between MRI and histological observations of perivascular edema and cellular infiltration (Quintana et al., 2006). This study supports these findings, showing a strong association between T_2_ signal intensity and Lm, as well as with increased immune cell infiltration.

Increased accumulation of leukocytes in the lung parenchyma and abnormal inflammatory responses is correlated with airflow limitation and decreased lung function (O'Shaughnessy, Ansari, Barnes, & Jeffery, 1997; Saetta et al., 1998; Suzuki et al., 2017; Wang et al., 2018). CS exposure alone significantly increased cellular infiltration foci in mouse lung parenchyma compared with controls. However, we also show that low‐dose azithromycin significantly reduced the number of these foci, which could contribute to the reduction in emphysematous changes seen in CS + Azi mice, and reiterate the anti‐inflammatory effects of this macrolide. Further studies are needed to elucidate the mechanisms underlying the immunomodulatory activity of azithromycin in emphysema and COPD.

This study demonstrates that interventional treatment with low‐dose azithromycin may attenuate emphysematous changes caused by chronic CS exposure in mice. Furthermore, these changes were detectable using in vivo imaging techniques, which correlated with histological analyses. To our knowledge, this is the first study to treat mice with low‐dose azithromycin following the development of a CS‐induced inflammatory lung environment; representative of administering treatment to smokers who have sought medical advice as their lungs begin to deteriorate.

Limitations of this study include a lack of mechanistic data, resulting from insufficient cell numbers in BAL. Future studies that more extensively investigate mucus hypersecretion (MUC5AC and MUC5B in the BAL), leukocyte subsets, neutrophil activation status, and soluble mediator production, focusing on some of the mediators that might be interesting in human COPD development (e.g., neutrophil elastase/other proteases) are warranted. Also worth investigating is the mechanism through which azithromycin could modulate emphysema development through alteration of the respiratory tract (or gut) microbiome.

Although further work is required, the findings of this study suggest that intervention with long‐term, low‐dose azithromycin treatment may offer therapeutic benefits to current smokers (particularly those who are attempting to quit) and patients with early pulmonary emphysema.
